# Supplementary material for: A nanomaterials discovery robot for the Darwinian evolution of shape programmable gold nanoparticles
Source: Nat Commun. 2020 Jun 2;11:2771. doi: 10.1038/s41467-020-16501-4 (PMC7265452; doi:10.1038/s41467-020-16501-4)
Supplement: Supplementary file 1 — Supplementary Information [file 41467_2020_16501_MOESM1_ESM.pdf]

Supplementary Information for:

Robotically Assisted Darwinian Evolution of Shape Programmable Gold nanoparticles

Daniel Salley† et al.

School of Chemistry, The University of Glasgow, Glasgow, G12 8QQ, UK.

*Correspondence to: Lee.Cronin@glasgow.ac.uk; http://www.croninlab.com

Table of Contents

Supplementary methods 3
General Experimental Remarks 3
Hardware 4
Platform Design and Preparation 4
Assembly of the wheel platform: 5
Full platform assembly 9
Fluidic connections 10
Software 12
Hardware Interfacing 12
Platform Control 12
Initialisation 12
Experimental 13
Analysis 13
Watcher 13
Genetic algorithm (GA) 14
Order of Execution 15
Algorithm 15
Fitness functions 17
Chemistry 19
Manual synthesis of gold nanoseeds. 19
Manual synthesis of gold nanorods. 19
Platform stock solutions 20
UV-Vis Analysis 21
Au Nanospheres 21
Au Nanorods 23
Expanded Search 25
Transmission Electron Microscopy (TEM) 27
Au nanospheres 28
Au Nanorods 29
Expanded Search Synthesis 30
Chemical handling 31
Code and data availability 31

Supplementary methods

General Experimental Remarks

Solvents and reagents were used as received from commercial suppliers unless otherwise stated. All 3D components were designed and exported from Onshape.com, a professional cloud-based CAD software and were printed using full cure720 RGB material from Stratasys on an Objet500 Connex. Structural v-slot aluminium rail and connective hardware were bought from Ooznest.com. All other non-electrical components were laser cut from 6 or 4 mm acrylic using a Monster laser ML1060 with a 130W CO_2 laser from Radecal Machine. UV-Vis spectra for experiments on the bench were collected using JASCO V-670 spectrometer in absorbance mode using quartz cuvettes with 1.0 cm optical path length. For the automated platform, UV-Vis spectra were recorded using a DH-2000-S light source and a flow cell FIA-Z-SMA 905 (10 mm path length) from Ocean Optics, connected by fibre optics to an Flame-S-VIS-NIR spectrometer. Transmission Electron Microscopy (TEM) images were recorded on FEI Tecnai T20 transmission electron microscope equipped with Gatan Imaging Filter.

Hardware

Platform Design and Preparation

All wheel structural components were custom designed on Onshape.com an open-source 3D design software online, and either printed on SYS Connex 3D printer or laser cut from acrylic (6 and 4 mm). A 15-vial circular tray, driven by a Geneva wheel mechanism was created to complete full generations of reactions in parallel. A bearing system mounted the driven wheel to the central column, secured to the base plate. An outer frame was constructed of aluminium v-bar from Ooznest.com and was used to protect the wheel and offer mounting positions for liquid handling hardware. A static dispensing position was mounted on the outer frame. Each vial was filled at this position using TriContinent C300 pump. The total dispensing time of 15 reaction vials was approximately 30 minutes, also the ideal time for stirring before examination by UV spectroscopy. Once vial 1 had reached position 15 it was removed via a modular syringe driver, designed in-house and controlled again by in-house software. Each solution was removed, examined by UV spectroscopy, the vial cleaned extensively at the position and the next reaction solution moved into position. The entire process of 15 reactions (one generation) can be completed in under an hour. The drive wheel rotation was produced by a Nema17 stepper motor, controlled via in-house software. Direct individual stirring was achieved via two 7.5 x 2 x 2 Nd bar magnets in a custom designed and 3D printed housing on top of any array of 12V DC fans, speed controlled via PWM via Arduino Mega 2560. The array was further mounted beneath the vial tray on 4 3D printed legs, mounted directly to the base plate of the platform. A step by step assembly of this platform can be seen in Supplementary Figure 1.

Assembly of the wheel platform:

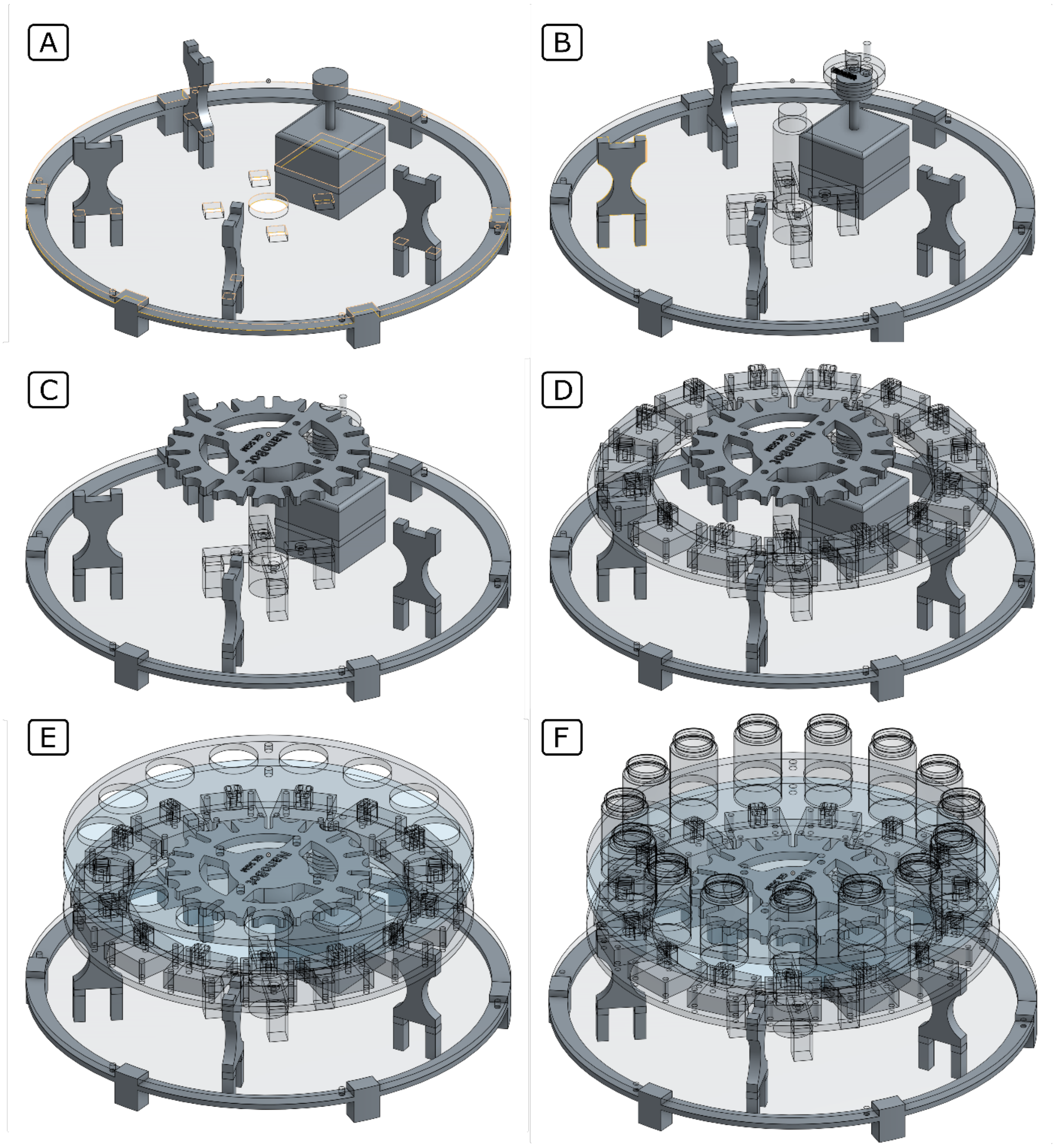

Supplementary Figure 1 Series of partial constructions of the wheel platform. **a)** Base print with acrylic plate with fan ring legs and a mock of the Nema17 motor **b)** Geneva driven wheel mounting column and Geneva drive wheel mounted on the stepper motor added **c)** 15 position driven wheel print added **d)** 15 x 12V DC 30x30 mm fans mounted on an acrylic cut ring with magnet housing attached added. **e)** Lower and upper 15 slot vial tray cut from 4mm acrylic mounted on the Geneva driven wheel added. **f)** Vial tray filled with 15 x 14mL reaction vials added.

All commercial components mentioned below can be found in the bill of materials section of the Github link provided. All STL files can be found in the same link in the 'prints' folder. 'Subassemblies' A-E here refer to component assemblies described in section 1.2.1.

- The acrylic laser cut base is secured to the 'NanoBot Base Platform' piece using up to six M3 x 12mm screws and nuts. **(**Supplementary Figure 1A)
- The Nema17 40 mm stepper motor, fitted with the pololu aluminium hub (Subassembly A) is fed from under the base ensuring the cables remain beneath the acrylic plate. **(**Supplementary Figure 1B**)**
- The four 'NanoBot stir ring supports' are fitted into the base. (Supplementary Figure 1B)
- The 'NanoBot Column Mount' is secured to the base with four M3 x 12 mm screws and nuts (Supplementary Figure 1B)
- The 'NanoBot drive wheel' is secured to the stepper motor via the aluminium mounting hub using two M3 x 16 mm screws and nuts (Supplementary Figure 1B)
- The 'NanoBot driven wheel' and 35mm OD bearing required (subassembly B). This subassembly is mounted on the 'NanoBot Column Mount' via this bearing. (Supplementary Figure 1C)
- You can now test the quality of the printed parts by manually turning the drive wheel to see its relation to the driven.
- Place Fan array (subassembly C and D) onto the four 'NanoBot stir ring supports'. (Supplementary Figure 1D)
- Remove the driven wheel assembly briefly and follow the instructions detailed in 'Vial tray' subassembly 4. Replace the complete assembly as before to complete the wheel platform. (Supplementary Figure 1E)
- Test the balance of the platform by adding fifteen 14 mL vials to the tray. (Supplementary Figure 1F)

Subassemblies

The following are subassemblies are those described in the above assembly instructions. A-E refer to the images shown in

A. Nema17 with Pololu aluminium hub:

1. Using the grub screw provided, secure the Pololu aluminium hub flush, to the top of the Nema17 stepper motor shaft at the flat face (Supplementary Figure 2A).

B. Driven wheel with bearing:

1. Press fit the 35mm OD bearing into the underside of the driven wheel (Supplementary Figure 2B)

C. Magnet housing

1. Each magnet housing consists of two 3D printed parts and two 15 x 4 x 4mm Nd magnets.
2. First, glue the ‘NanoBot magnet housing base’ piece to the ***centre*** of the mobile side of a 25 x 25mm DC fan.
3. Second slide both magnets into the slots of the ‘NanoBot magnet housing 2.2kg pull rectangular’ 3D print opposing each other. The magnets should push each other away into the out walls of the slots (Supplementary Figure 2C).
4. Finally, place the ‘NanoBot magnet housing 2.2kg pull rectangular’ containing the magnets onto the glued ‘NanoBot magnet housing base’ on top of the fan.

D. Stirring fan array:

1. Secure using M3 x 20mm screws and nuts, fifteen subassembly C units to the acrylic cut ring. It is crucial to consider the wiring of the fans; the cables can be extended to ensure they can reach neatly around the ring to be combined at one point for connection to the Arduino board PWM later. (Supplementary Figure 2D)

E. Vial tray/driven wheel:

1. Remove the driven wheel temporarily from the assembly.

2. Secure the acrylic cut vial tray base to the top of the driven wheel using M3 x 12/16mm screws.
3. Feed three M3 x 30 mm screws from the underside of the vial tray base, through the hollow 'NanoBot vial tray hex spacer' and the top vial tray acrylic piece. Secure each screw with an M3 nut to complete the vial tray. (Supplementary Figure 2E)

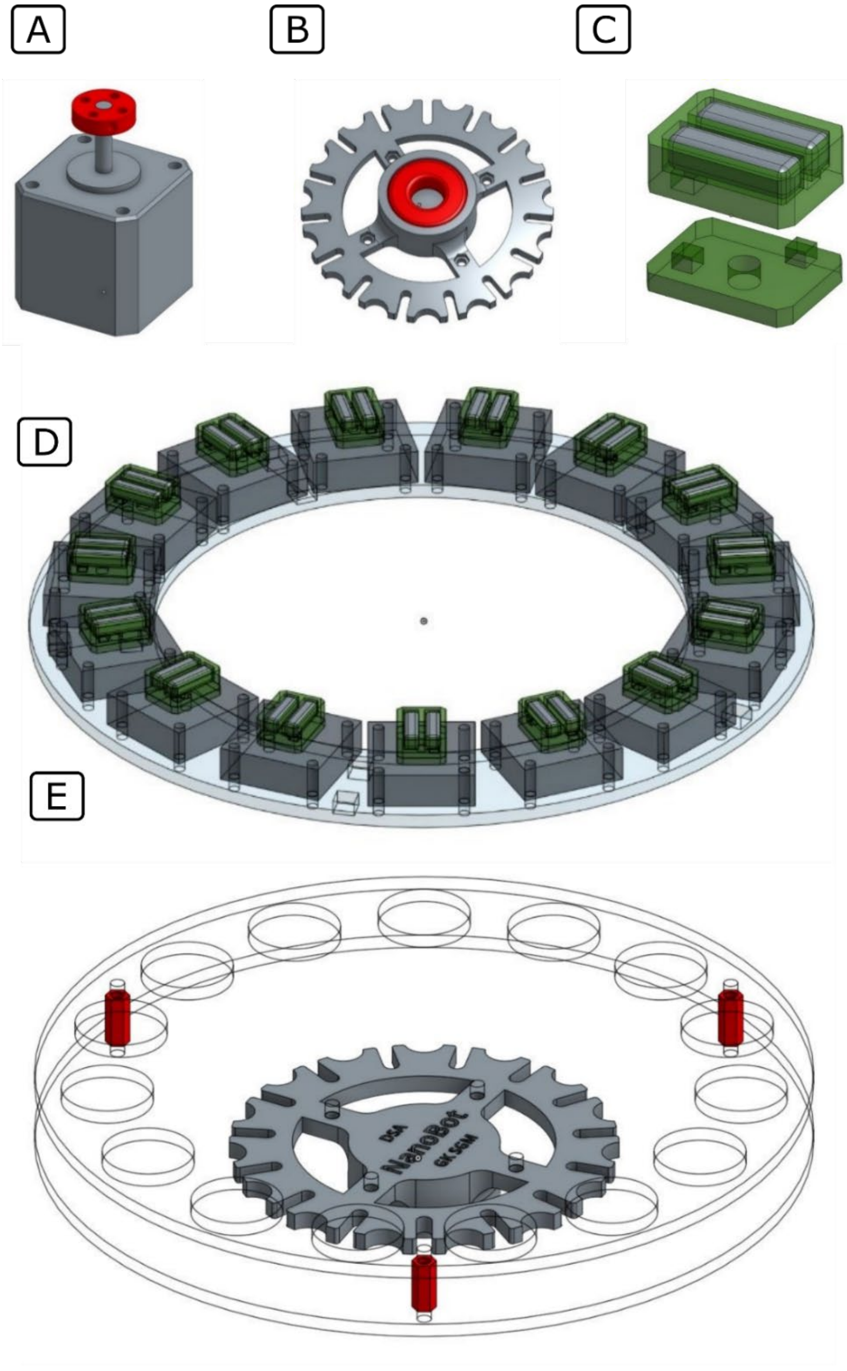

Supplementary Figure 2 Subassemblies of robotic platform

Full platform assembly

Supplementary Figure 3 shows both the virtual and actual constructed platform.

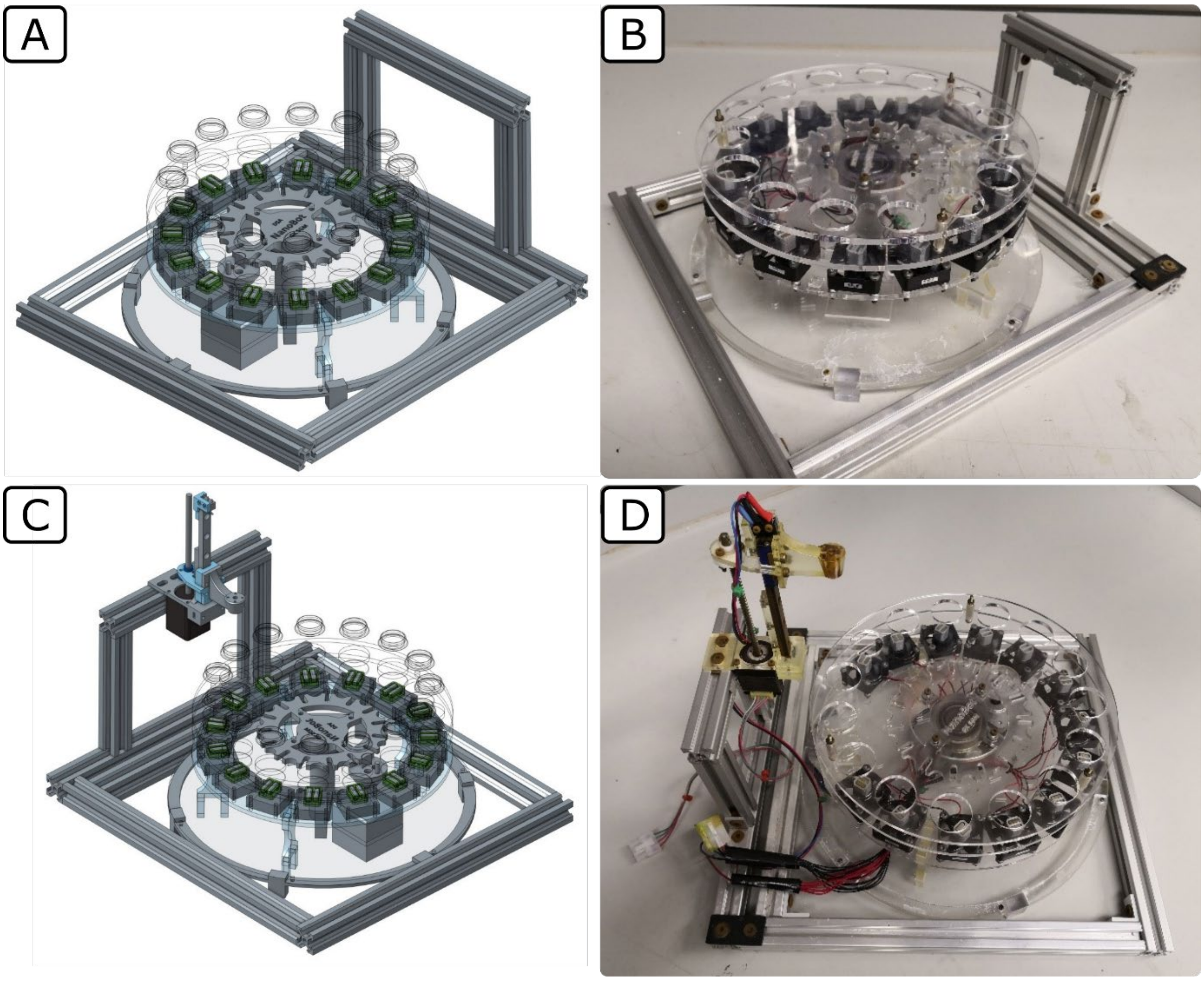

Supplementary Figure 3 Complete platform with surrounding frame and liquid handling additions. a) V-bar aluminium outer frame added. b) Static dispensing position secured over position/reaction vial 1 and modular syringe driver for sample removal and vial cleaning mounted over position/vial 15 added completing the system in full.

Fluidic connections

(NB links to all parts in the following section can be found in bill_of_materials.md file on the Github repository)

Tri-continent c-series syringe pumps were used exclusively on this platform. A variety of syringe sizes are used from 1-12.5 mL depending on pumps assigned function (see liquid handling section).

1.6mm OD and 3.2mm OD PTFE Kinesis/Cole-Palmer tubing was used, again depending on the function assigned to the pump. Tube connections to the pump valve outlet/inlets were made by IDEX flangeless fittings Nat PP 1/16 in or 1/8 in depending on the tube. The tube to dispensing needle connections are made up of three pieces: first the tube is secured into another IDEX flangeless fitting (Supplementary Figure 4A), the fitting is then screwed into a Restek Thames 1/4-28 female to male Luer asy (Supplementary Figure 4B) and finally the Adhesive Dispensing (AD5125TLC) 25-gauge TLC lined tip needle (Supplementary Figure 4C) is secured to the internal threads of Luer. Using the parts listed is crucial as after much trial and error, these were the units found to be chemically compatible with some of the harsh reagents used in this study. Supplementary Figure 4shows this assembly of fluidic connections. Part A was also used to connect to the valve of each tricontinent C-series pump

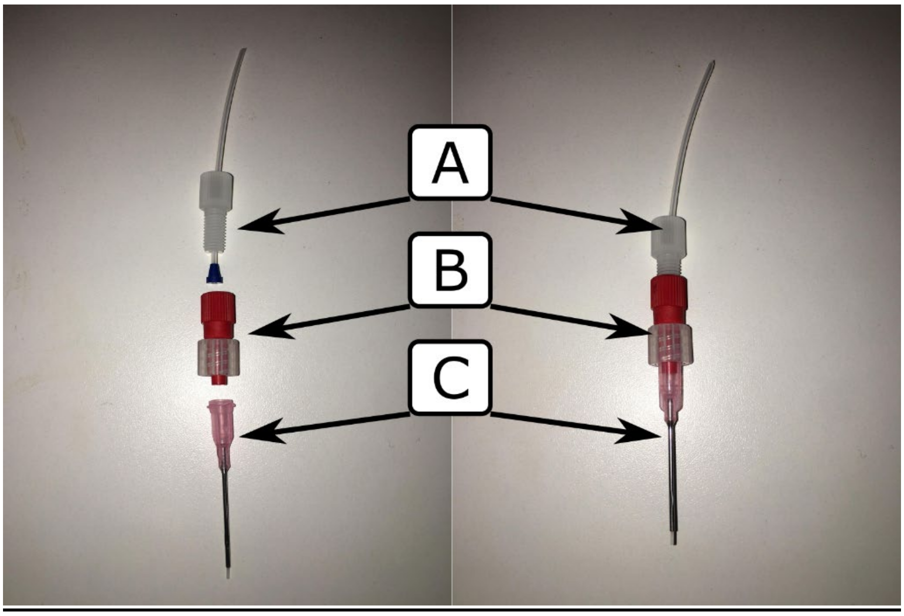

Supplementary Figure 4 Exploded and assembled views of the fluidic connections used for this work.

The full experimental set-up can be seen in Supplementary Figure 5 below.

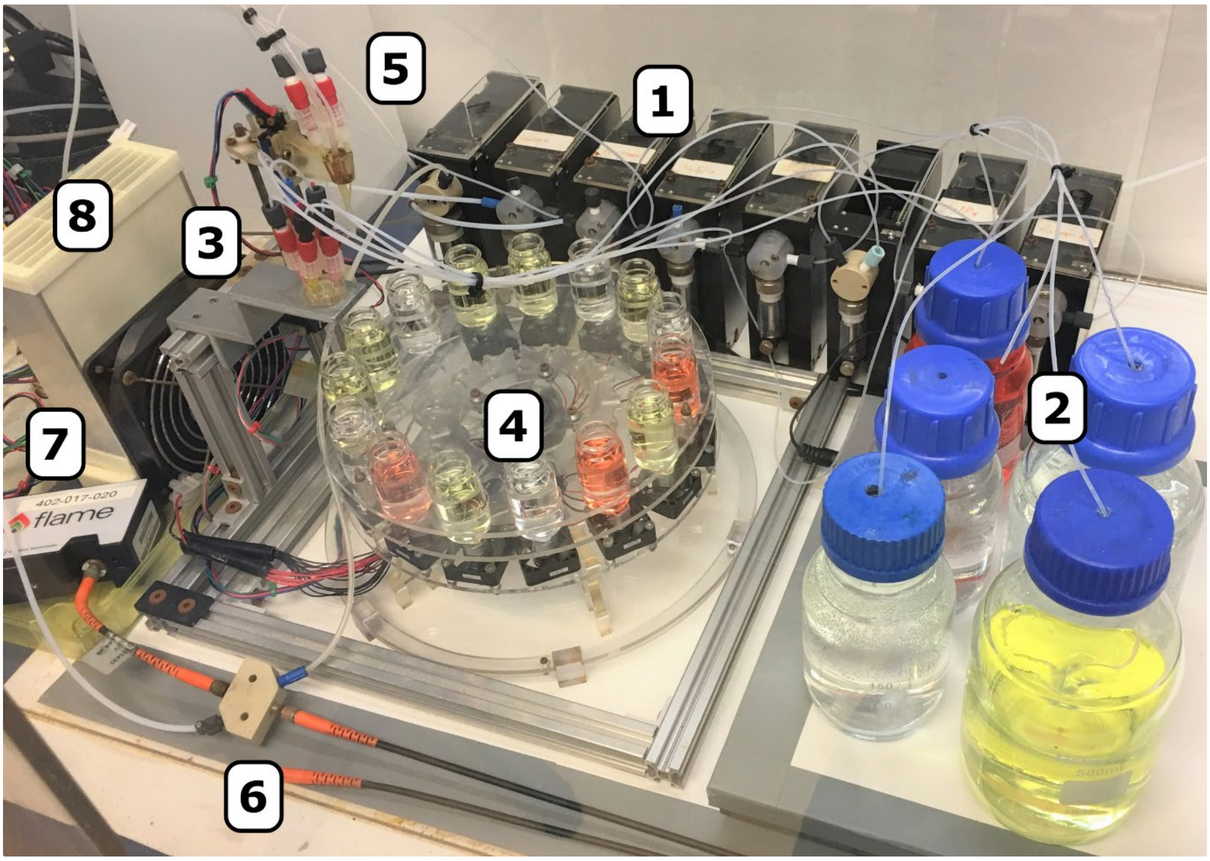

Supplementary Figure 5 Set up used for the robotically assisted evolution of gold nanoparticles. 1) Tri-continent C3000 syringe pumps 2) Reagent bottles on stirring plate 3) Dispensing stage 4) Geneva wheel with vial tray 5) Sample extraction module 6) Flow cell/optics 7) Ocean optics flame UV-Vis spectrometer 8) Heating element. Pumps are connected to the reagents in order to be dispensed into the different vials placed in the Geneva wheel using the dispensers. Samples are analysed using UV-Vis analysis after the designated reaction time. Heater is used in order to keep the temperature constant inside the box where the platform is placed.

Software

Hardware Interfacing

All aspects of the platform were controlled via software developed in-house using Python 2.7/3.6. The Tricontinent C3000 pumps were controlled through a python library via the PC serial port. All moving hardware including stirring arrays, controllable sample station, and the Geneva wheel were controlled via python interfacing with an Arduino Mega 2560 board (C++). UV measurements were obtained through development of a python wrapper class on top of an external library for OceanOptics devices (https://github.com/ap--/python-seabreeze).

Platform Control

The software to run the platform was divided into three distinct sections:

- Initialisation
- Experimental
- Analysis
- Watcher
- GA

Initialisation

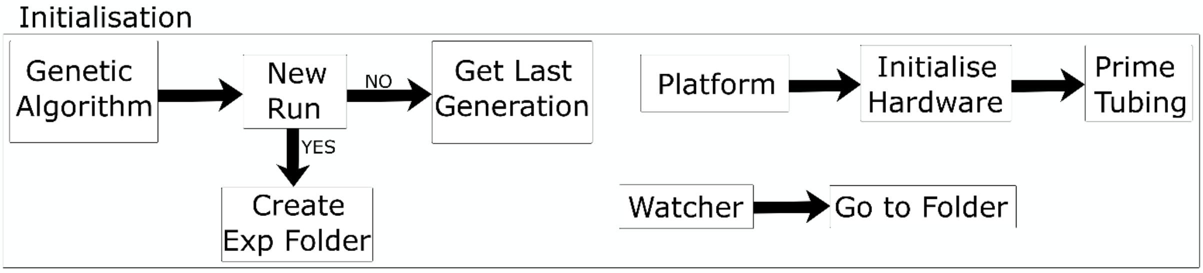

Supplementary Figure 6 Initialisation sequence for platform control

The user runs the execution script, passing in the command line arguments of the numeric ID of the experiment and the number of generations they wish to run. This will generate a folder for the experiment and a corresponding information file detailing the genetic algorithm parameters and number of generations. Then, the platform will initialise the hardware. (Supplementary Figure 6).

Experimental

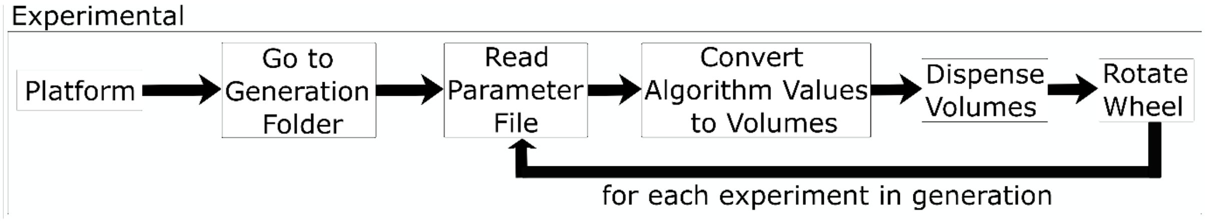

Supplementary Figure 7 Platform experimental sequence

When a generation folder has been created, the script will then run through each experiment folder within, reading the parameters files and converting the algorithm values to volumes. The system will dispense the volumes and rotate the wheel. This process will be repeated as many times as experiments per generation (15 times in our case)(Supplementary Figure 7)

Analysis

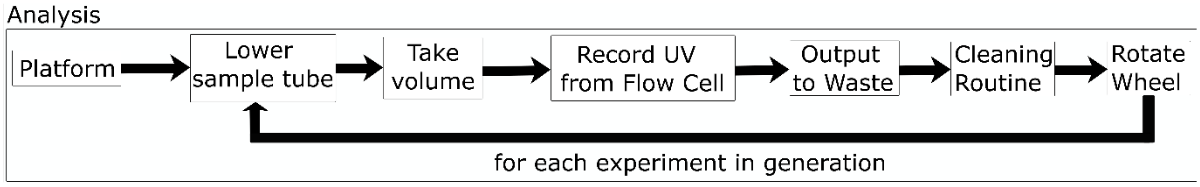

Supplementary Figure 8 Analysis sequence for a given sample

Once all experiments have concluded, the system lowers the sample tube and takes a specified volume for in-line UV analysis and the contents disposed of. A cleaning cycle starts and when it finishes the wheel rotates. This process repeats until all generations have been exhausted. (Supplementary Figure 8)

Watcher

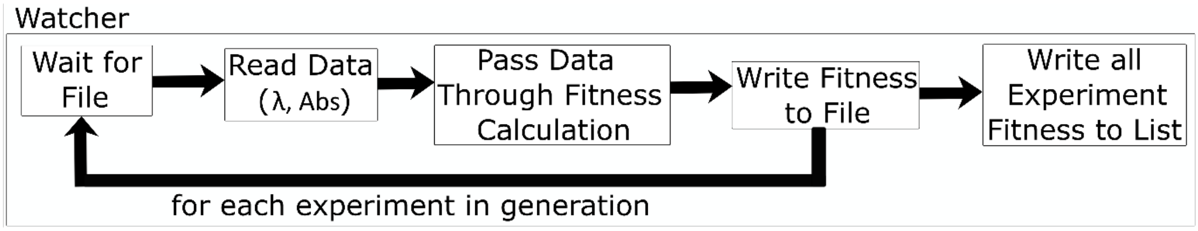

Supplementary Figure 9 The sequence of the 'Watcher' program overseeing all process during an experimental series

The user runs this script, passing in the command line arguments of the type of experiment (spheres/rods) etc. and the numerical ID. The script will then “listen” in each experiment of a generation

for specific files. First, it will check if a parameter file exists, signalling a valid experiment. It will then check for a file containing the raw UV data from the spectrometer. This file is parsed and processed, outputting an image of the spectrum and a file containing the fitness of the data. This process is repeated as many times as experiments per generation. (Supplementary Figure 9).

Genetic algorithm (GA)

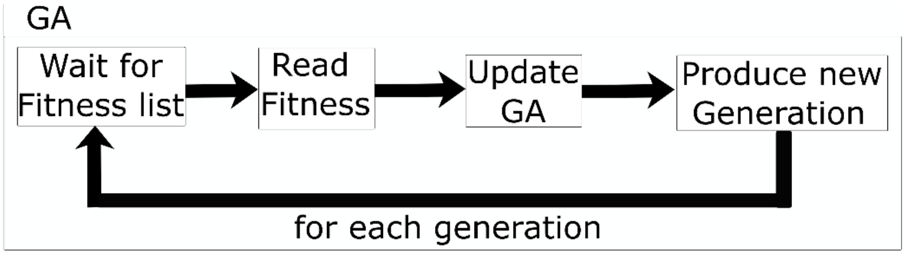

Supplementary Figure 10 GA sequence for a reaction generation

Once the experimental and analysis script have concluded with the generation and the fitness of each experiment is evaluated, these values are gathered in a single file and passed into the genetic algorithm. This then leads to the creation of a new generation folder with updated experimental parameters reflecting the updates from the algorithm (Supplementary Figure 10). The process repeats until the end of the experimental run (Supplementary Figure 11)

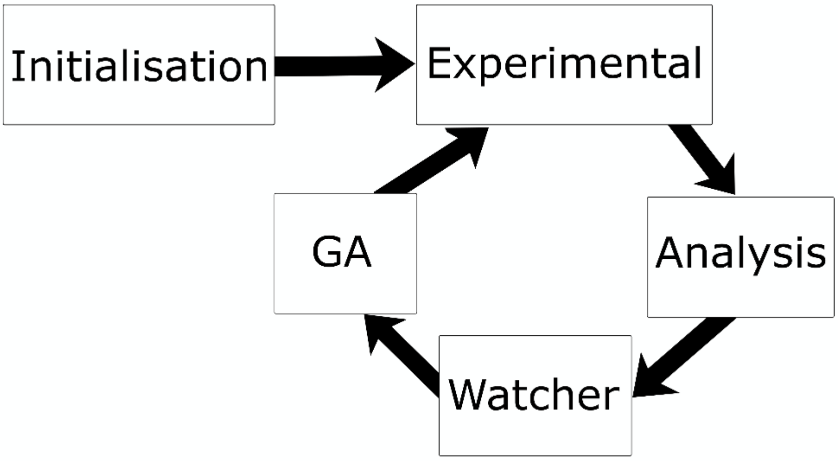

Supplementary Figure 11 Explanatory diagram of the functioning of the platform. Initialisation only happens when the user switches on the platform and starts the experiments, then, it starts an iterated cycle where the first script is the experimental, followed by the analysis, the watcher and the GA

Order of Execution

The order of execution is as follows:

- Run the execution script, passing in command line arguments
- Wait for a confirmation prompt from the execution script
- Run the generation creator script, passing in command line arguments
- Run the data processing script, passing in command line arguments
- Trigger the prompt on the execution script
- Experiments commence

Algorithm

The algorithm used within this system was a standard optimisation genetic algorithm – a form of evolutionary algorithm. The way the genetic algorithm proceeded was by starting with a set of random solutions and carried out the evaluation of their outcomes based on their fitness factor. In this case, the evaluation is based on the UV-Vis spectra of the samples. Depending on the fitness factor values, the system started the selection and recombination process. Selection is usually with replacement, which means that outcomes with better fitness factor values will have more chances to be selected. After the selection, the algorithm recombines the parameters of the solutions, creating a new set of solutions. This process is iterated, the fitness factor values improve until it reaches a certain criterion.

The process can be divided into the following six steps:

- **Initialisation:** An initial set of randomly generated parameters based on a random seed. All generated arbitrary values are between the range of 0 and 1 which are then normalised to mL totalling 10 mL.
- **Evaluation:** Analysis of the fitness values of each experiment.
- **Selection:** Decides on what solutions will survive onto the next generation by placing copies of those solutions into the next generation. This imposes a "survival of the fittest" mechanism on the solutions. 4 survivors are selected for survival from one generation to the next. The 4 survivors are sampled probabilistically with a probability proportional to their fitness function

after a SoftMax transformation is applied with a temperature coefficient of 0.1 and without replacement, meaning that the same individual cannot be selected twice for survival.

- **Recombination (generating the remaining list of new reactions after survivor selection):** Combination of parts of two or more of the selected "parent" solutions to create new and potentially better solutions (offspring). This is achieved through the "one-point" method - takes one point of each of the parent's formulation and split it, then the different parts of the formulation from each parent are merged and create a new formulation for the offspring. The specific details of the generation of the remaining individuals are as follows:
    - Two parents are sampled probabilistically with a probability proportional to their fitness function after a softmax transformation is applied with a temperature coefficient of 0.1. From the parents, the child is generated by:
        - Cross over of the parent's genome (see genome_crossover()), for each gene in the genome, the child gene will be computed following these rules:
            - 25% of the time, the child inherits entirely from parent 1
            - 25% of the time, the child inherits entirely from parent 2
            - 50% of the time the child genome is a weighted average of parent1 and parent2 genome. The mixing weight is sampled randomly and uniformly within the range [0, 1]
- **Mutation:** Randomly modifies a solution (made of individual values, each a 'genome') whilst recombination occurs. One or more traits of an individual are modified, leading to a "random walk" in the direction of a candidate solution. From this genome, we apply a mutation with a per locus rate of 0.3 and a per locus SD of 0.1, meaning that:
    - Each gene has a 30% probability of being mutated

- If a gene is mutated, its value is shifted by adding a number sample from a Gaussian probability distribution with 0 mean and 0.1 standard deviation.

- **Replacement:** Offspring created through selection, recombination, and mutation replace their parental counterparts.
- **Termination:** End of experiment.

The process of **Evaluation** through to **Replacement** cycles continuously, creating a new generation for each iteration (See Supplementary Figure 11). This repeats until a threshold of generations, defined by the experimenter, has been met. These actions are summarized in Supplementary Figure 12.

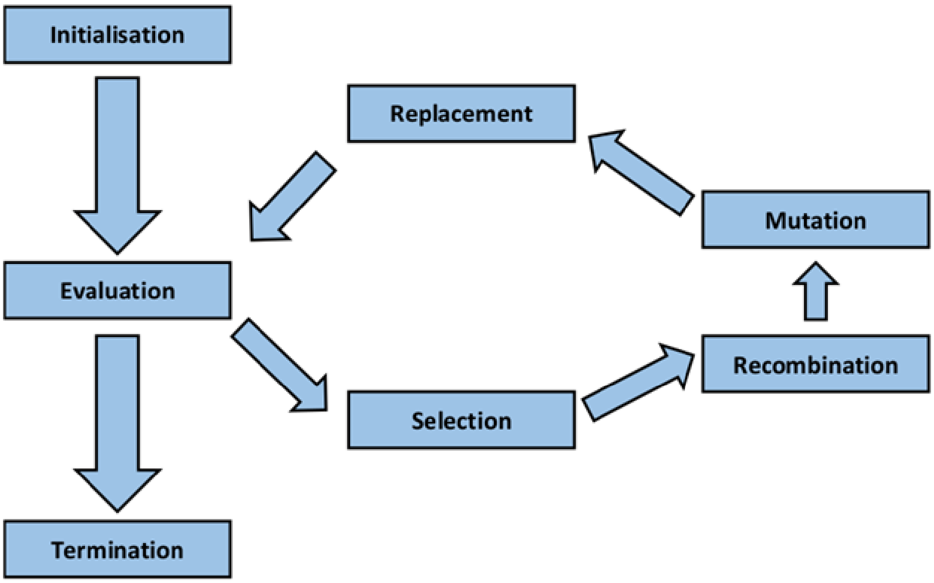

Supplementary Figure 12 Explanatory diagram of genetic algorithms. Evaluation, selection, recombination, mutation and replacement are iterated through generations. Initialisation and termination only happen once.

Fitness functions

The following section describes individual fitness functions used in this work to produce gold nanospheres, rods and octahedrons (variable names and terms are found in figure 4 of the manuscript):

- **Space 1, spheres:** Space one was concerned with achieving a single peak spectrum of 553 nm from simple seeds and chemicals. Two parameters were considered: Position of the λmax value

of the reaction in question vs the objective and the absorbance value at which the experimental data intersected the objective peak. Normal distributions were generated for the objective and experimental λmax value. Observed/Target yielded the first value of the function between 0-1. This handled the peak position component. The method of differentiating yield i.e. absorbance level between samples was the handled by the second term, $Abs_χ$. Simply adding the absorbance value at 553 nm (target peak position) of the experimental data to the first term yield a maximum fitness of 2.6, accounting for all relevant spectra features.

- **Space 2, Rods:** A given spectra was divided into 3 regions a (490-560 nm), b (580-640 nm) and c (700-800 nm). As rods between 40-60 nm begin to form, ideally region a would grow in terms of absorbance slightly, b would minimise, and c would grow substantially. For each spectra the area of each of these regions beneath the absorbance curve would be calculated. The simple calculation to determine the fitness of a sample toward our desired goal was: Ac / (Ab+Aa). This simple calculation drove the system to produce fitness values above 5 for many samples and provided the optimised synthesis seen in **manuscript table 1** and the seeds for space 3.
- **Extended space:** To produce a single peak outcome using rods as seeds we again used a method similar to space 2. The peak we wished to produce was 580 nm and so the region x was 560-640 nm. The region in which we wished to eradicate all spectra features was 640 nm onwards. Region y was set therefore at 640-740 nm. Again, the area of each region was calculated for a given sample and A_x/A_y determined its fitness. This simple calculation encouraged to system to grant higher yielding samples, higher fitness as well as optimise for a specific λmax value.
- All fitness values were subject to absorbance thresholds and penalties for the detection of multiple peaks.

Chemistry

As explained in the manuscript, each three levels of hierarchical evolution involved seed mediated synthesis of gold nanoparticles. Each level involved multiple generations of 15 reactions each, attempting to move toward a predetermined/desired UV spectrum. In each case this process began with a generation of random reaction reagent ratios (no prior knowledge) based on a numerical seed. Using the data from these reactions the algorithm would learn the reaction space by applying fitness values to each of the experimental spectra based on its similarity to the target. Over time and successive generations, the platform would move closer to the desired target spectra.

Following are the general synthesis and stock solutions used during the three levels of evolution.

Manual synthesis of gold nanoseeds.

For the synthesis of gold nanoseeds there were needed 3 reagents, which were $HAuCl_4$, CTAB and $NaBH_4$. The synthesis method followed for spherical nanoseeds, was the one described by Nikoobakht.[18] This method describes the synthesis of seeds as mixing CTAB solution (5 mL, 0.2 M), with $HAuCl_4$ (5 mL, 0.0005 M) and to this mixture a solution of ice cold $NaBH_4$ (0.6 mL, 0.01 M) under vigorous stirring. The reaction was carried out at 30 °C, stirred for 5 minutes and left undisturbed for 30 minutes before being diluted. These seeds were used in space 1 and 2 for the synthesis of gold nanospheres (AuNSs) and gold nanorods (AuNRs)

Manual synthesis of gold nanorods.

(particles needed to obtain the spectral target of AuNRs for the platform) The synthesis of gold nanorods follows the next procedure: CTAB (5 mL, 0.2 M) were added to a solution with $AgNO_3$ (0.15 mL, 0.004 M) and HAuCl4 (5mL, 0.001 M). After gentle mixing, ascorbic acid (70 µL, 0.0788 M) were added and the colour of the solution became colourless. The last step was the addition of 12 µL of gold seeds. The solution was kept at constant stirring and at 30 °C for 30 minutes, left for 60 minutes to grow and examined by UV.

Platform stock solutions

Level 1: Spheres

- Hexadecyltrimethylammonium bromide >99 % Sigma-Aldrich, CTAB molecular biol (0.2 M)
- Gold(III) chloride trihydrate 99.9 % Sigma-Aldrich, HAuCl4 (0.001 M).
- Ascorbic acid, 99.9% Sigma-Aldrich, (0.00575 M)
- Au seeds (above), synthesis above (10.6 mL total) diluted to 30 mL. This was to ensure accurate volume transfer from syringe pumps

Level 2: Rods

- Hexadecyltrimethylammonium bromide >99 % Acros Organics, CTAB (0.2 M)
- Gold(III) chloride trihydrate 99.9 % Sigma-Aldrich, HAuCl4 (0.001 M).
- Silver nitrate, 99.9999 % Sigma-Aldrich, $AgNO_3$ (0.00005 M)
- Ascorbic acid, 99.9% Sigma-Aldrich, (0.0065 M)
- Au seeds (above), synthesis above (10.6 mL total) diluted to 30 mL. This was to ensure accurate volume transfer from syringe pumps

Level 3: Extended octahedral producing space:

- Hexadecyltrimethylammonium bromide >99 % Acros Organics, CTAB (0.2 M)
- Gold(III) chloride trihydrate 99.9 % Sigma-Aldrich, HAuCl4 (0.001 M).
- Silver nitrate, 99.9999 % Sigma-Aldrich, $AgNO_3$ (0.00005 M)
- Ascorbic acid, 99.9% Sigma-Aldrich, (0.0065 M)

AuNR seeds (from optimized synthesis of Level 2 synthesis). 24 x 10 mL identical reactions of the highest optimised rods from space 2 were performed. Each was centrifuged at 12,000 RPM, washed, combined and dispersed to 240 mL total.

UV-Vis Analysis

The following spectra (Supplementary Figure 13 to Supplementary Figure 16) are from three complete algorithm driven experimental runs to optimise for spherical, rod shaped and octahedral shaped particles from simple starting materials. Experiments from early to final generations are included to see the progress made by the algorithm toward the desired target run. These UV have corresponding TEM analysis in section 5.

Au Nanospheres

Supplementary Figure 13 Shows the progression across 10 generations of reactions toward an AuNS target of 553 nm

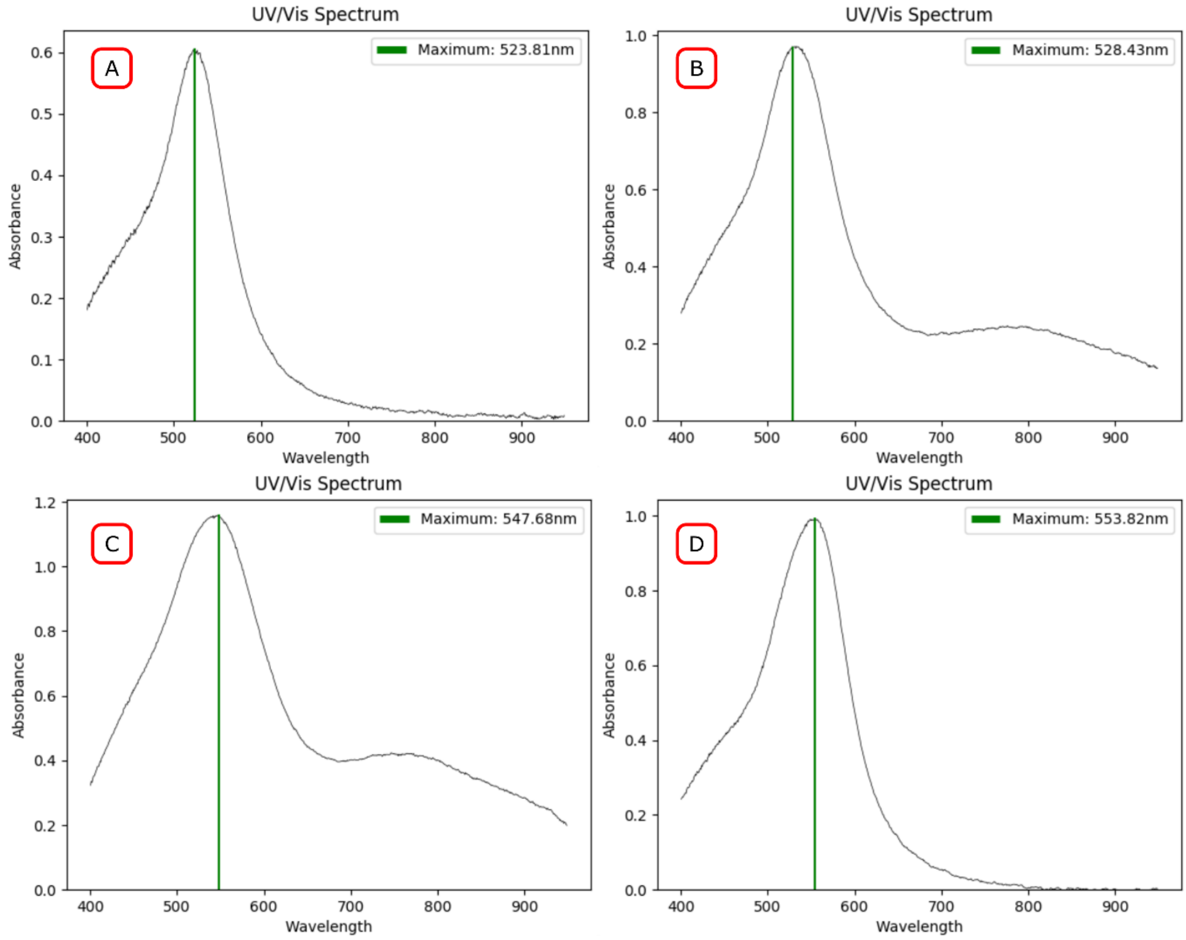

Supplementary Figure 13 Image Spectra of generation 1 (random selection) showing low absorbance of likely 10-15 nm particles. Image B spectra from generation 4 showing improved absorbance and peak location still significantly far from target (553 nm). Image C spectra from generation 8 showing

an example of particles close to the target with an unfortunately broadened signal. Image D generation 8 showing the target peak achieved with a sharp well-defined signal.

The reaction conditions that produced spectra Supplementary Figure 13 A-C (D included in main text) can be seen in Table 1:

**Spectra**	**CTAB (mL)**	**$HAuCl_4$ (mL)**	**Ascorbic acid (mL)**	**≈ 2 nm Seeds (mL)**
**A**	0.078	3.328	6.321	0.371
**B**	2.243	5.324	2.257	0.018
**C**	0.676	6.12	2.71	0.49

Supplementary Table 1 Reagent combinations that produce the UV signals seen in Supplementary Figure 13.

Au Nanorods

Supplementary Figure 14 Shows the progression across 10 generations of reactions toward an AuNR target

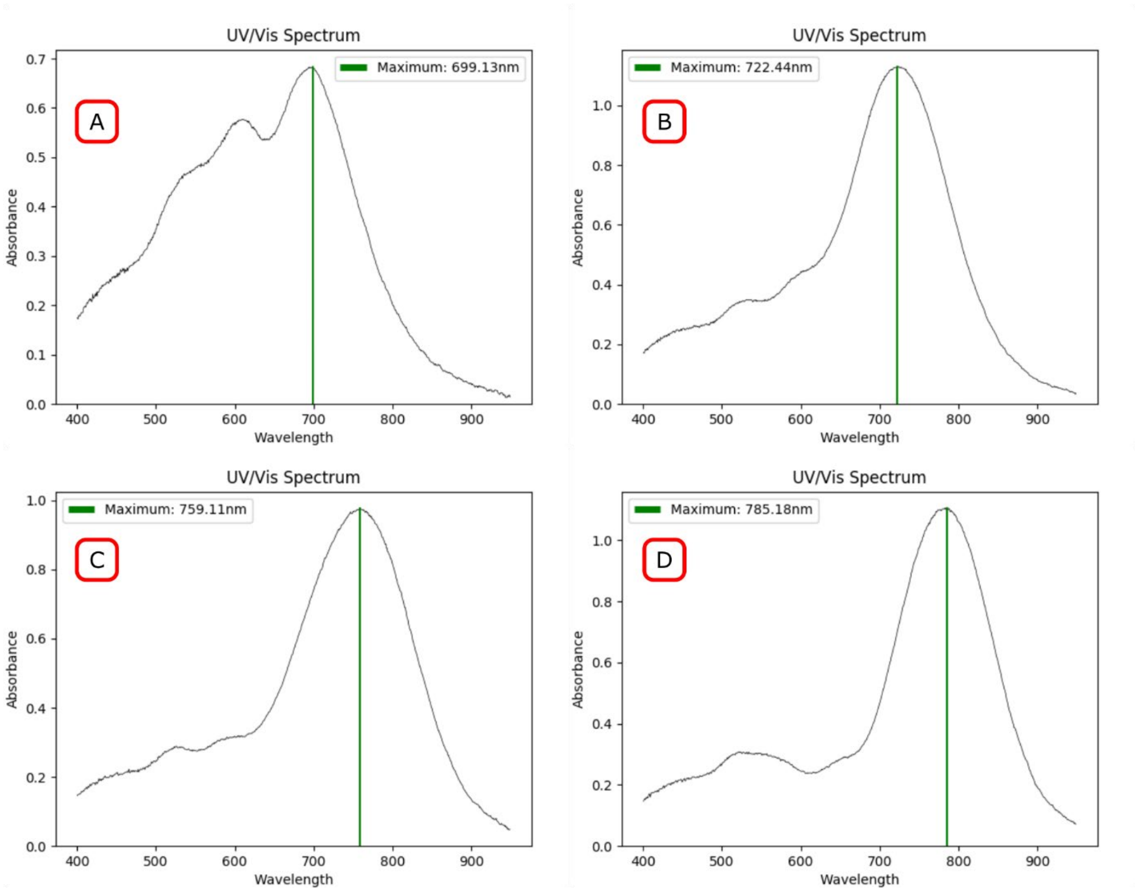

Supplementary Figure 14 Spectra of generation 1 (random selection) showing multiple signals of unknown mixture. Image B spectra from generation 4 showing the two desired peaks with a feature contamination at approx. 605 nm. Image C spectra from generation 5 showing clear progress of the longitudinal peak position with far less 605 nm contamination. Image D generation 10 showing an experiment with good resemblance to the target peaks with the feature at 605 nm all but eradicated.

The reaction conditions that produced spectra Supplementary Figure 14 A-C (D included in main text) can be seen in Table 2:

Spectra	CTAB (mL)	$HAuCl_4$ (mL)	Ascorbic acid (mL)	$AgNO_3$ (mL)	≈ 2 nm Seeds (mL)
**A**	2.404	3.01	2.147	2.378	0.06
**B**	3.512	2.717	1.889	1.821	0.06
**C**	4.276	2.31	1.848	1.505	0.06

Supplementary Table 2 Reagent combinations that produce the UV signals seen in Supplementary Figure 14.

Expanded Search

For this generation run the ≈ 2 nm seeds were replaced with a large suspension of the optimised rods produced in space 2. This involved performing 24 identical reactions on the platform using the reagent ratios the system provided. The 24 x 10 mL reactions were centrifuged at 12,000 RPM, washed and resuspended in 240 mL type 1 ultra-pure water (same cumulative volume as the original reactions). These particles were subjected to no other purification processes i.e. all particles produced using these conditions were present in the 240 mL stock. Each reaction should have produced rods with a 780 nm longitudinal peak, once combined the solution returned the following spectrum (Supplementary Figure 15)

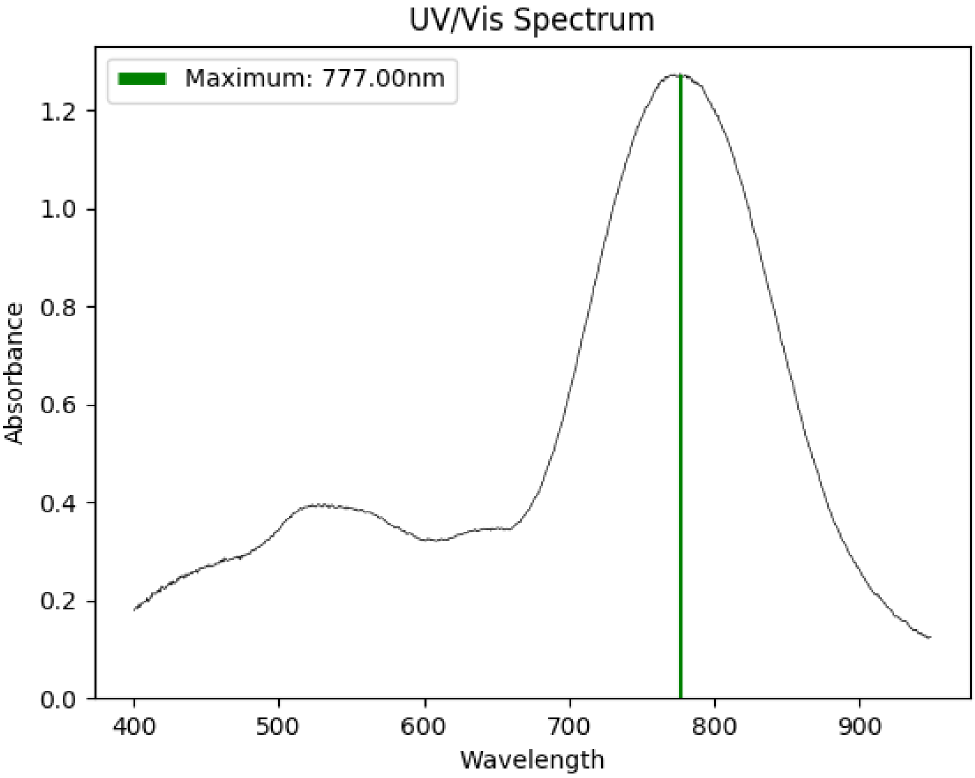

Supplementary Figure 15 Spectrum of 24 combined rod reactions using the synthesis provided by the GA driven series of reaction generations seen in section 5.2.

Supplementary Figure 16 Shows the progression across 8 generations of reactions toward a Spectral target of 580 nm

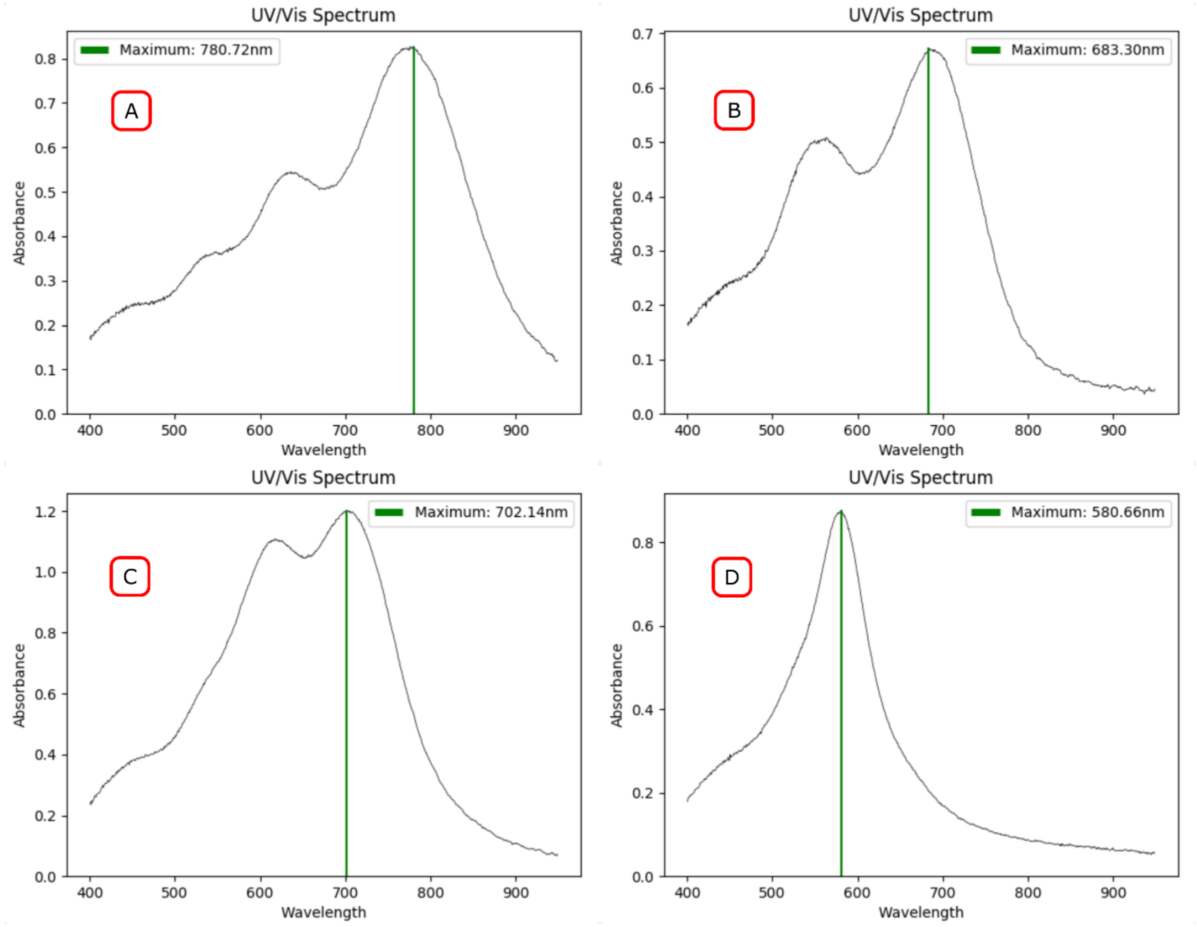

Supplementary Figure 16 Image Spectra from generation 1 (random selection) showing clearly that much of the rod seed has survived with the strong absorbance at 780 nm however some reactivity has taken place given the extra feature at 620 nm. Image B spectra from generation 2 shows a clear shift of the longitudinal peak of the seed from 780 nm to 683 nm, the peak at ≈ 550 nm also indicates growth along the transverse edge of the particles. Image C shows significant change to the original seed, producing near merging of the spectra features into a single peak indicating a progression toward a substantial increase in particle symmetry. Image D generation 8 showing the highest scoring sample compared to target peak. This peak is sharp and high absorbing with no identifiable contaminant features.

The reaction conditions that produced spectra A-C (D included in main text) can be seen in Table 3:

Spectra	CTAB (mL)	$HAuCl_4$ (mL)	Ascorbic acid (mL)	$AgNO_3$ (mL)	Rod Seeds (mL)
**A**	3.23	2.296	1.848	1.625	1
**B**	2.147	1.149	3.59	2.114	1
**C**	0.44	4.593	2.914	1.053	1

Supplementary Table 3 Reagent combinations that produce the UV signals seen in Supplementary Figure 16.

Transmission Electron Microscopy (TEM)

An experiment, which demonstrated ideal UV spectra, was chosen to be repeated in bulk on the platform. The products were centrifuged at 10-12,000 RPM to obtain the nanoparticles and taken for TEM analysis. The following images were taken from experiments synthesised on the platform. None of the samples were purified beyond CTAB removal by centrifuge, the images are representative of the complete solution populations.

Au nanospheres

Supplementary Figure 17 Shows the progression from simple to make, 10-15 nm AuNSs toward to the final target of 80 nm spheres over the course of 10 generations.

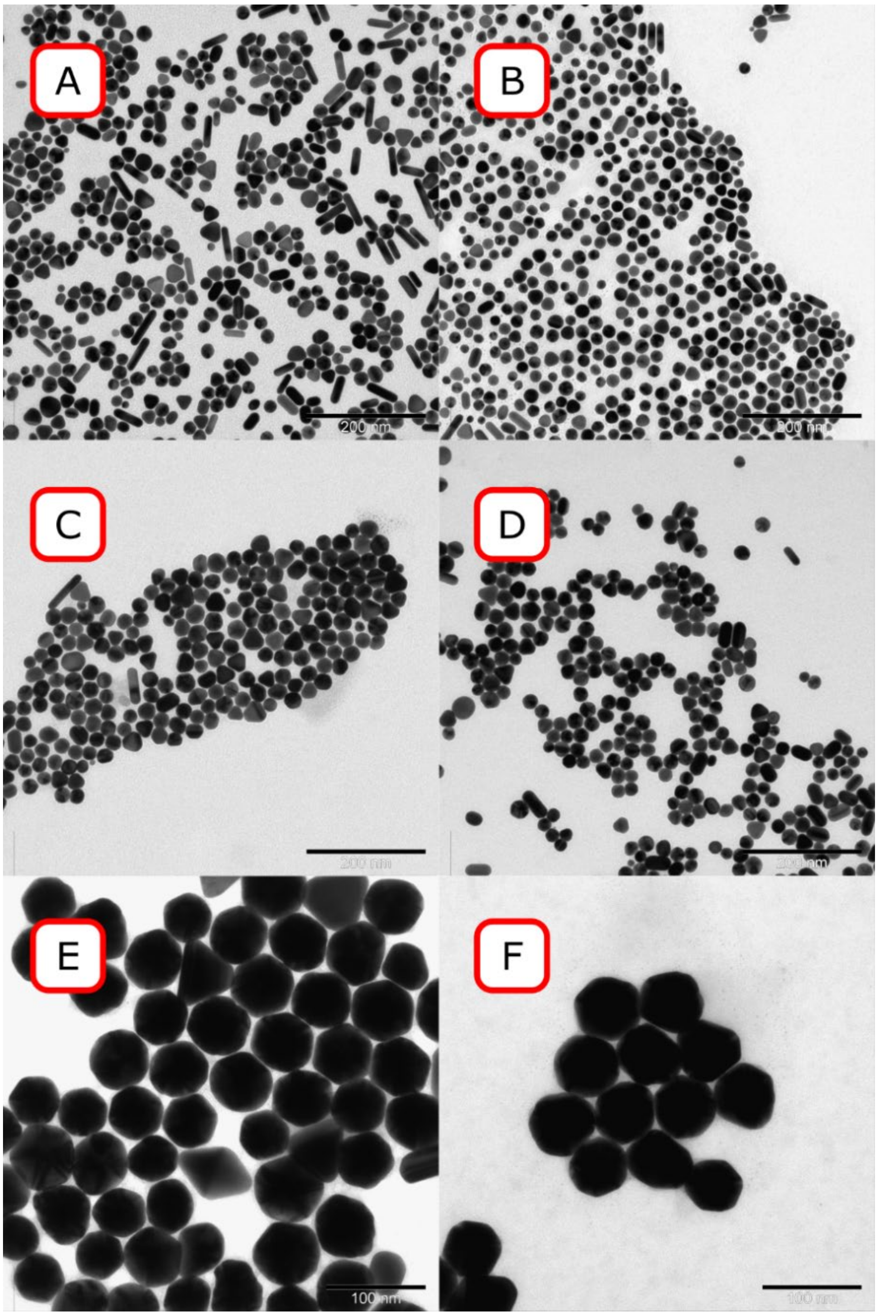

Supplementary Figure 17 TEM images of spheres from the early, middle and final generations of the series showing progression toward desired shape. [A] 10-15 nm mixture of spheres, small rods and triangles, generation 4 (corresponding to UV [A] in Supplementary Figure 13). [B] 10-15 nm spheres with minimal by-products, generation 7 (corresponding to UV [A] in Supplementary Figure 13). [C] 15-25nm spheres with minimal by products, generation 8 (corresponding to UV [B] in Supplementary

Figure 13). [D] 20-40nm spheres from Generation 7 (corresponding to UV [C] in Supplementary Figure 13). [E and F] 70-80nm spheres from Generation 9 (corresponding to UV [D] in Supplementary Figure 13).

Au Nanorods

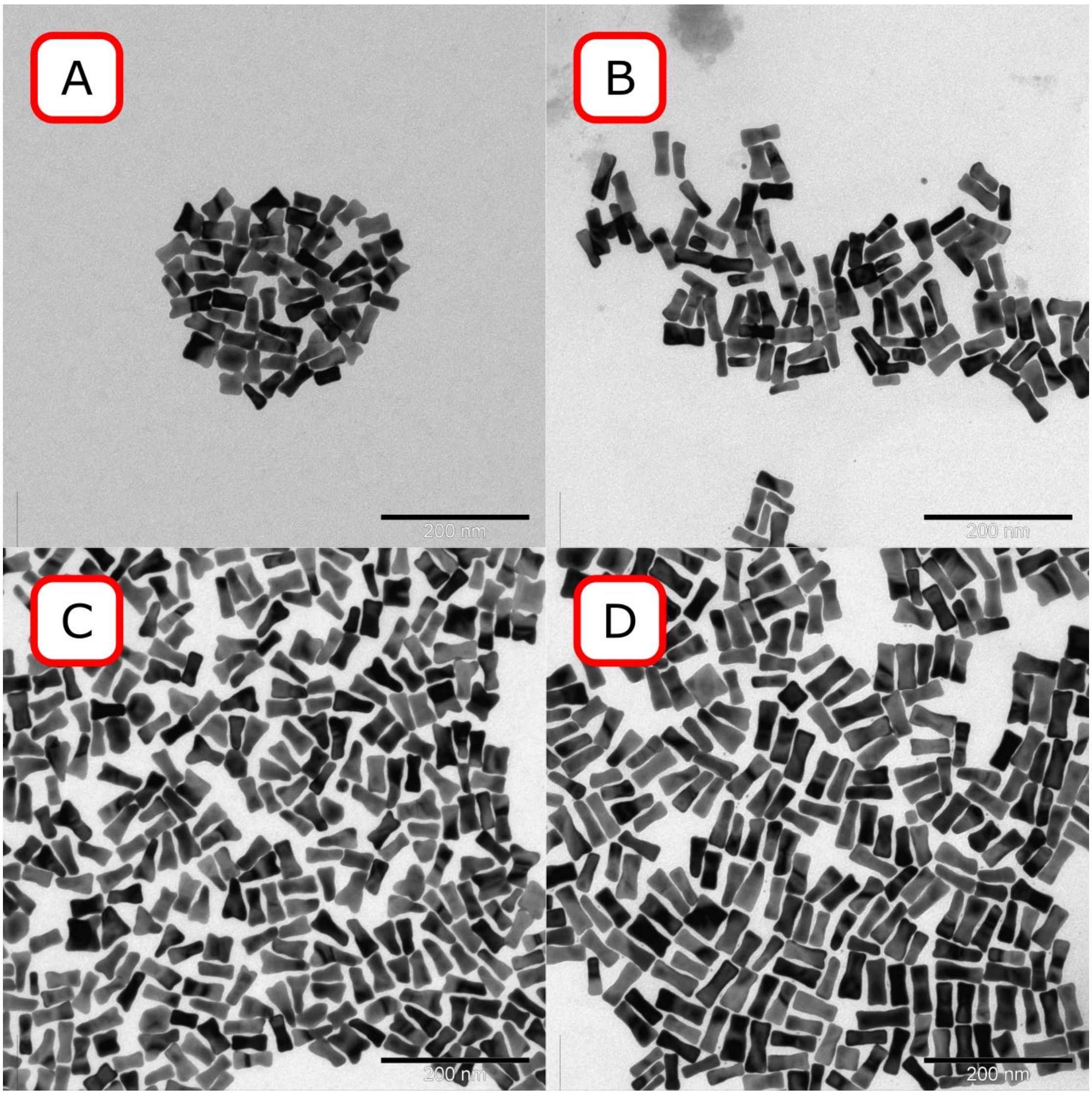

Supplementary Figure 18 TEM images of the particles obtained from the early, middle and final generation using the automated system. [A] Distorted bone-shaped particles, non-uniform with multiple undefined shapes from generation 1 (corresponding to UV [A] in Supplementary Figure 14). [B] Low yielding, improved uniformity particles however still inadequate from generation 4 (corresponding to UV [B]in Supplementary Figure 14). [C] High yielding rod-like particles with ill-defined edges and

cubic/4-point star contaminants from generation 5 (corresponding to UV [C] in Supplementary Figure 14). [D] Final generation highest AuNRs showing good uniformity with minimal contaminants (corresponding to UV [D] in Supplementary Figure 14). These final particles were those used as seeds for the expanded space search. The synthesis is robust and repeatable using the system.

Expanded Search Synthesis

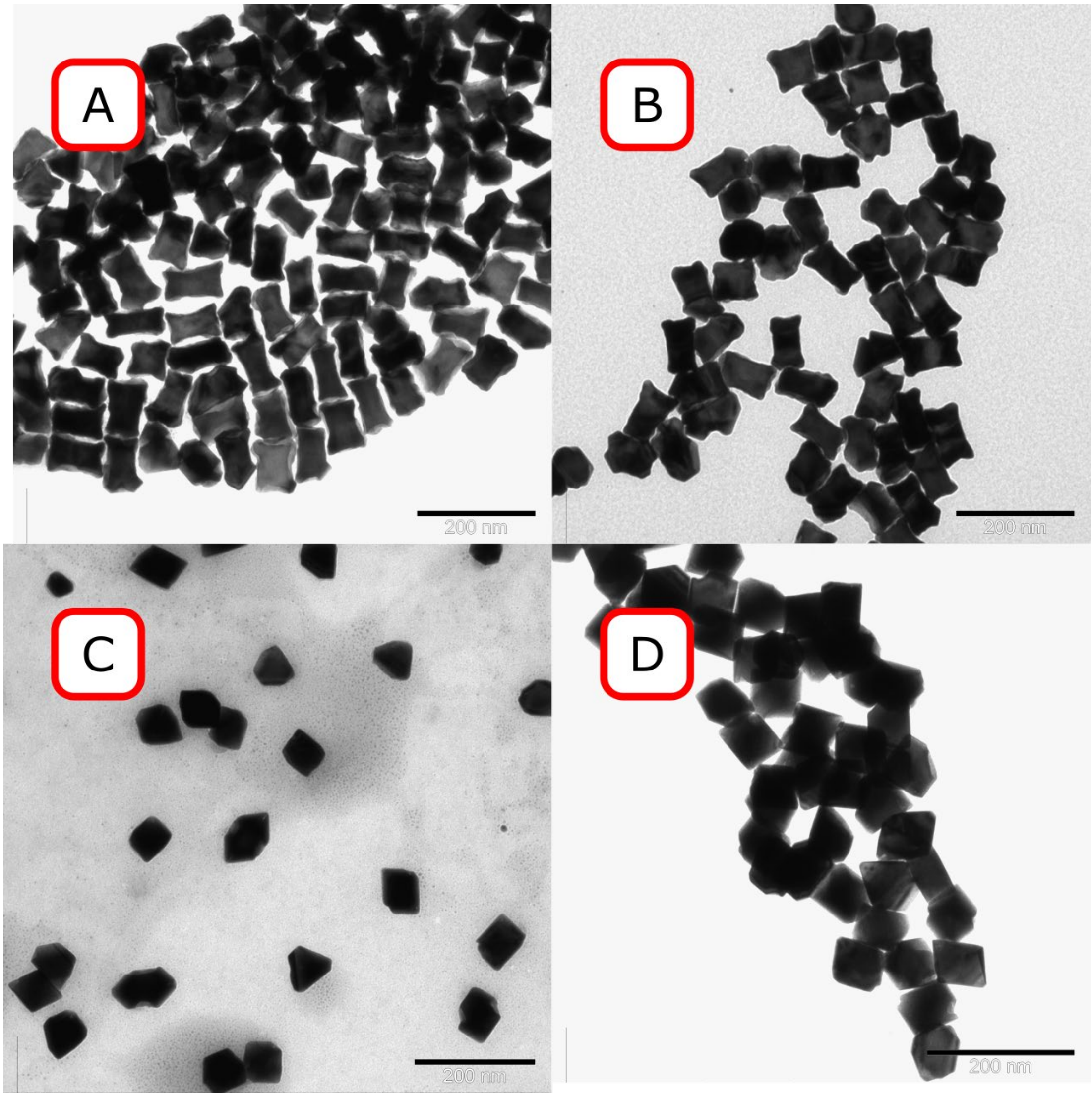

Supplementary Figure 19 TEM images of the progression toward the eventually found octahedral particles (580 nm single peak spectra) obtained using the automated system. Image [A] shows a small level of growth on the rod edges but little change to the particles (corresponding to UV spectra [A] in Supplementary Figure 16). Image [B] shows an increase in this growth and further distortion of the rod seed particles (corresponding to spectra [B] in Supplementary Figure 16). Image [C] shows a substantial

change to the original seed, showing several arrow-headed particles alongside some completely symmetrical octahedrons (corresponding to UV spectra [C] in Supplementary Figure 16). Image D shows the highest fitness sample found by the automated system revealing almost full conversion of the rod seed to symmetrical octahedral nanoparticles (corresponding to spectra [D] in Supplementary Figure 16 having acquired the target spectra).

Chemical handling

All chemicals were supplied by *Fisher Chemicals* and *Sigma Aldrich* and were used without further purification. Solutions were freshly prepared before each experiment. PTFE tubing with different internal diameters were supplied by *Kinesis* (Kinesis Ltd.).

After preparation, solutions were stored in glass bottles and stored at the required temperature (30 °C in all the cases except for the $NaBH_4$ and the ascorbic acid that were kept at ice cold temperature). The reagents were connected to the TriContinent™ pumps equipped with 5 mL or 1 mL syringes (depending on the reaction conditions) using PTFE tubing with internal diameter of 0.8 mm. The reaction mixtures were pumped through the UV-Vis flow cell through PTFE tubing with internal diameter of 2 mm and disposed to a 10 L waste drum using the same type of tubing. The cleaning cycles followed by the platform using water and acetone used PTFE tubing with internal diameter of 2 mm. The waste was disposed following the standard procedure for transition metal waste.

Code and data availability

Software used to perform this work can be found here:

https://github.com/croningp/NanomaterialsDiscovery

Data available upon request
